# Metabolic and functional imaging employment in the differentiation of brown tumors from bone metastases in a case of primary papillary thyroid cancer and parathyroid adenoma: case report

**DOI:** 10.1186/s43046-021-00089-x

**Published:** 2021-10-18

**Authors:** Ula Al-Rasheed, Fareed Barakat, Akram Al-Ibraheem

**Affiliations:** 1 Department of Nuclear Medicine, King Hussein Cancer Center, Amman, Jordan; 2 Department of Pathology and Lab medicine, King Hussein Cancer Center, Amman, Jordan

**Keywords:** Brown tumor, Hyperparathyroidism, Fluorine-18-2-fluoro-2-deoxy-d-glucose (^18^F-FDG), ^99m^Technetium sestamibi, Radioactive iodine (I-131) therapy (RAI)

## Abstract

**Background:**

Brown tumors are benign osteoclastic bone lesions encountered in patients with hyperparathyroidism. These tumors may demonstrate aggressive, destructive features in the skeleton and imitate metastatic bone lesions, particularly in patients with known primary neoplasm. In this case report of recurrent papillary thyroid cancer and ectopic parathyroid adenoma, we shed light on the importance of combining different nuclear medicine imaging modalities to differentiate brown tumors from metastatic bone lesions.

**Case presentation:**

We present a 39-year-old woman with a known history of papillary thyroid carcinoma classic type stage pT1N1b post-total thyroidectomy and radioactive iodine (I-131) therapy (RAI) presented with upper limb weakness and pain. An expansile lytic lesion involving the 6th cervical vertebra was seen in cervical spine MRI, which was suspicious for metastatic deposit. Therapeutic and diagnostic I-131 whole-body scans were negative for any I-13-avid lesions. Laboratory results revealed high calcium, parathyroid hormone, and alkaline phosphatase. A Technetium-99m-sestamibi (Tc-99m MIBI) scan was done with the standard protocol of spot views to the neck and upper chest area to localize any suspicious parathyroid adenoma. The scan demonstrated right supraclavicular and mediastinal Tc-99m MIBI-avid lesions suspicious for being ectopic parathyroid adenomas. Whole-body fluorine-18-2-fluoro-2-deoxy-d-glucose (^18^F-FDG), positron emission tomography/computed tomography (PET/CT) (^18^F-FDG PET/CT) was performed for further evaluation. It demonstrated multiple focal lytic skeletal lesions of abnormal increased FDG uptake as well as right supraclavicular FDG-avid lymph nodes. However, the superior mediastinal lesion was non-FDG-avid, suggesting the existence of two different entities: ectopic parathyroid adenoma with multiple brown tumors and metastatic right supraclavicular lymph nodes. The patient underwent right neck dissection and superior mediastinal mass excision. An intra-operative fresh serum parathyroid sample was sent, which dropped down to 100ng/ml from 863.7ng/ml. Later, histopathological results revealed that the right supraclavicular lymph nodes were metastatic papillary thyroid carcinoma. At the same time, the superior mediastinal mass proved to be parathyroid adenoma by histopathology, confirming the ^18^F-FDG PET/CT findings.

**Conclusions:**

In the case of papillary thyroid carcinoma, metastatic lymph nodes with hyperparathyroidism, and evidence of lytic bone lesions, careful interpretation of the different metabolic and functional imaging modalities are needed to exclude the concurrent parathyroid adenoma and facilitate the differentiation of brown tumors from bone metastases, leading to appropriate surgical and medical treatment plans.

## Background

Brown tumors are benign osteoclastic bone lesions encountered in patients with hyperparathyroidism due to the high level of serum parathyroid hormone (S.PTH). Brown tumors have a slightly greater frequency in primary than in secondary hyperparathyroidism (3% versus 2%). However, secondary hyperparathyroidism is much more common than primary hyperparathyroidism; therefore, most brown tumors that are seen are associated with secondary hyperparathyroidism. Brown tumor comprises multinucleated giant cells, spindle-shaped cells in a background of fibrous tissue [1].

Brown tumors may affect any bones in the body with different clinical presentations, overlapping with the presentation of bone metastases or polyostotic primary bone tumors [1]. Knowledge of clinical presentations, imaging features, and laboratory results is critical to differentiate these different diseases. In computed tomography (CT) scans, brown tumor features are not specific and may demonstrate multiloculated well-defined cystic lesions. The multiplicity and focality of brown tumor bony lesions may be wrongly interpreted on CT scan as multiple metastases and giant cell tumor (GCT) among other polyostotic primary bone diseases [1].

Brown tumors occur more in women with predilection in their 40s or 50s [2]. Brown tumors result from long-standing primary hyperparathyroidism that increases the osteoclast activity resulting in more lysis of the bone and eventually fibrous cortical cysts with hemosiderin deposits. Though brown tumors are benign, they may express high fluorodeoxyglucose (FDG) uptake, potentially due to giant cells in these lesions and intracellular macrophages [3]. Brown tumors are resolved as the PTH level goes back to normal after adequate parathyroidectomy [4].

Technetium-99m-sestamibi (Tc-99m MIBI) early and delayed imaging detects the lesions in parathyroid glands based on different washout rates between thyroid and parathyroid phases. It has an 85–95% accuracy rate in detecting pathologic parathyroid glands and plays an important role in preoperative localization. Zhao et al. reported that 27.8% of patients with hyperparathyroidism had bone uptake of MIBI, and the uptake was correlated with the level of PTH. The uptake mechanism in Tc-99m MIBI may be due to bone marrow fibrosis caused by excessive secretion of PTH. Bone mineral density scans show that most patients with hyperparathyroidism had osteoporosis due to the excessive secretion of PTH. This excessive secretion causes higher bone resorption than bone formation, leading to the loss of bone minerals. Metabolic bone disease can be detected by bone scintigraphy and is associated with high S.PTH and alkaline phosphatase (ALP) levels. In bone scintigraphy, Tc-99m MDP is chemically adsorbed and organically bound [5].Bone-seeking radiopharmaceuticals adsorb onto bone at sites of new bone formation, with a particular affinity for areas where active mineralization is occurring.

In our case report, we try to prove that the use of different metabolic and functional imaging modalities enables the diagnosis of concurrent presentation of parathyroid adenoma and PTC metastatic lymph nodes and facilitates the differentiation of brown tumors from bone metastases, resulting in an appropriate management approach.

## Case presentation

**Fig. 1:**
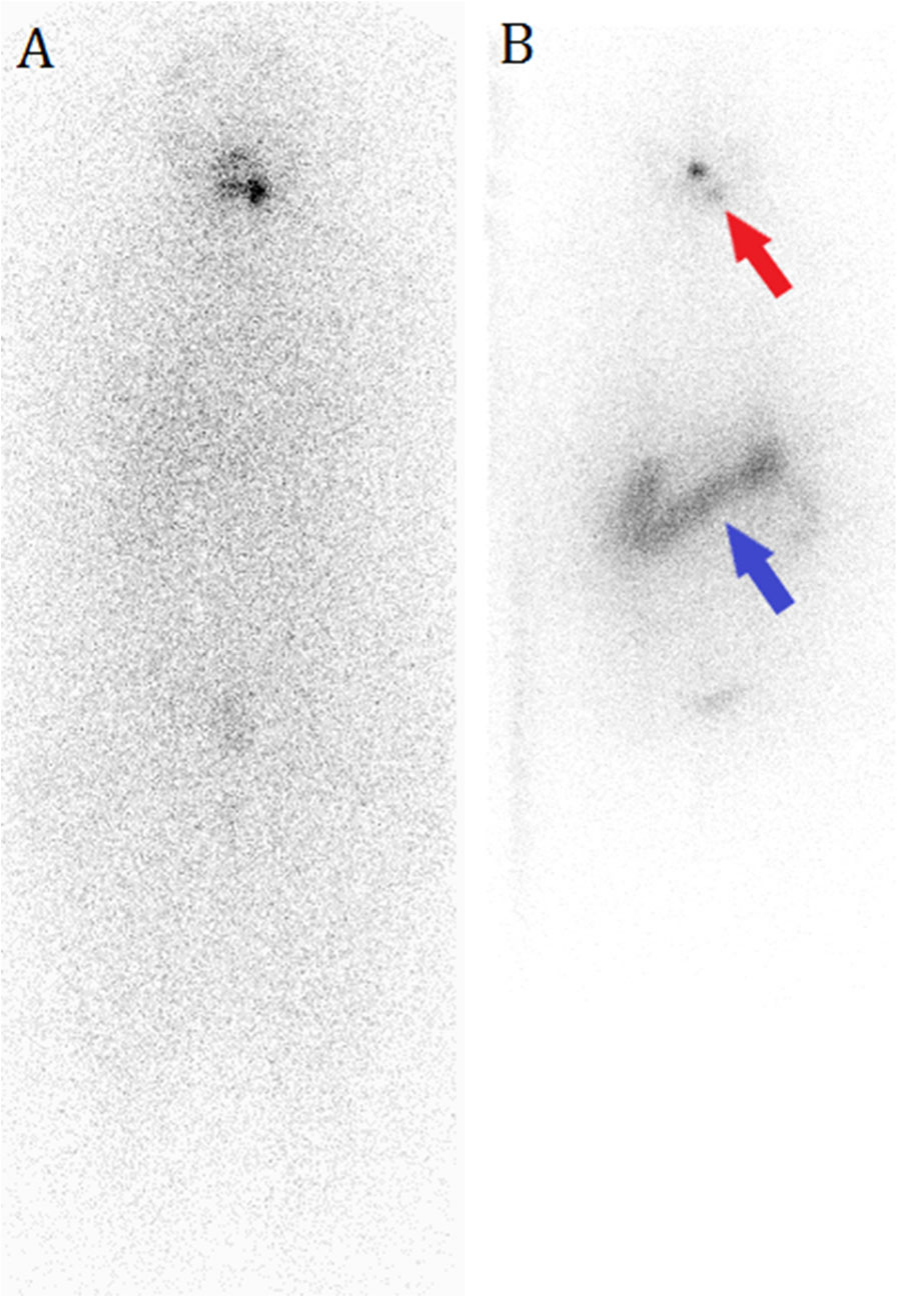
Whole-body scan by therapeutic RAI (**A**) and diagnostic RAI (**B**): Normal physiological distribution of the radioactive iodine including physiological uptake in the bowel (blue arrow) and salivary gland (red arrow), with no concerning radioactive iodine-avid pathologic uptake

We present a 39-year-old woman with a known case of papillary thyroid carcinoma classic type stage pT1N1b status, post-total thyroidectomy, and radioactive iodine therapy (^131^RAI). The patient presented 5 months after the ^131^RAI treatment with upper limb weakness and pain. An expansile lytic lesion involving the 6th cervical vertebra was seen in cervical spine magnetic resonance imaging (MRI), which was suspicious for metastatic deposit. Post-^131^RAI therapeutic and diagnostic scans were negative for any RAI-avid lesions (Fig. 1). Laboratory results revealed high stimulated serum thyroglobulin (sTg) level (145.7), high calcium (12.2 mg/dl), PTH (863.7ng/ml), and ALP (417 IU/ml).

**Fig. 2:**
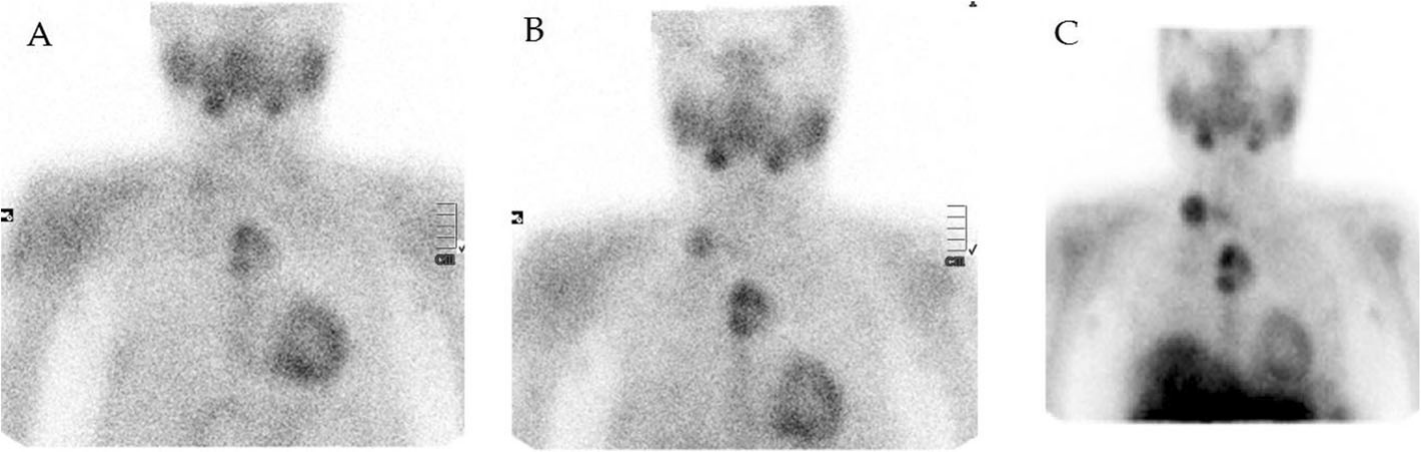
^99m^Tc MIBI scan images: **A** 20-min, **B** 1-h, and **C** 2-h images showing increased accumulation of Tc-99m MIBI uptake in the right supraclavicular and superior mediastinal areas

A Tc-99m MIBI scan was done 8 months later; planner images of the neck and chest were obtained at 20 min, 1 h, and 2 h. The images revealed a localized persistent right supraclavicular and mediastinal radiotracer retention, suspicious for ectopic parathyroid adenomas rather than metastatic papillary carcinoma (Fig. 2).

At the same time, the patient developed left wrist pain and swelling, for which a left wrist MRI was done. It showed a sub-articular eccentric lytic osseous lesion within the distal left ulna, suggesting a GCT.

**Fig. 3:**
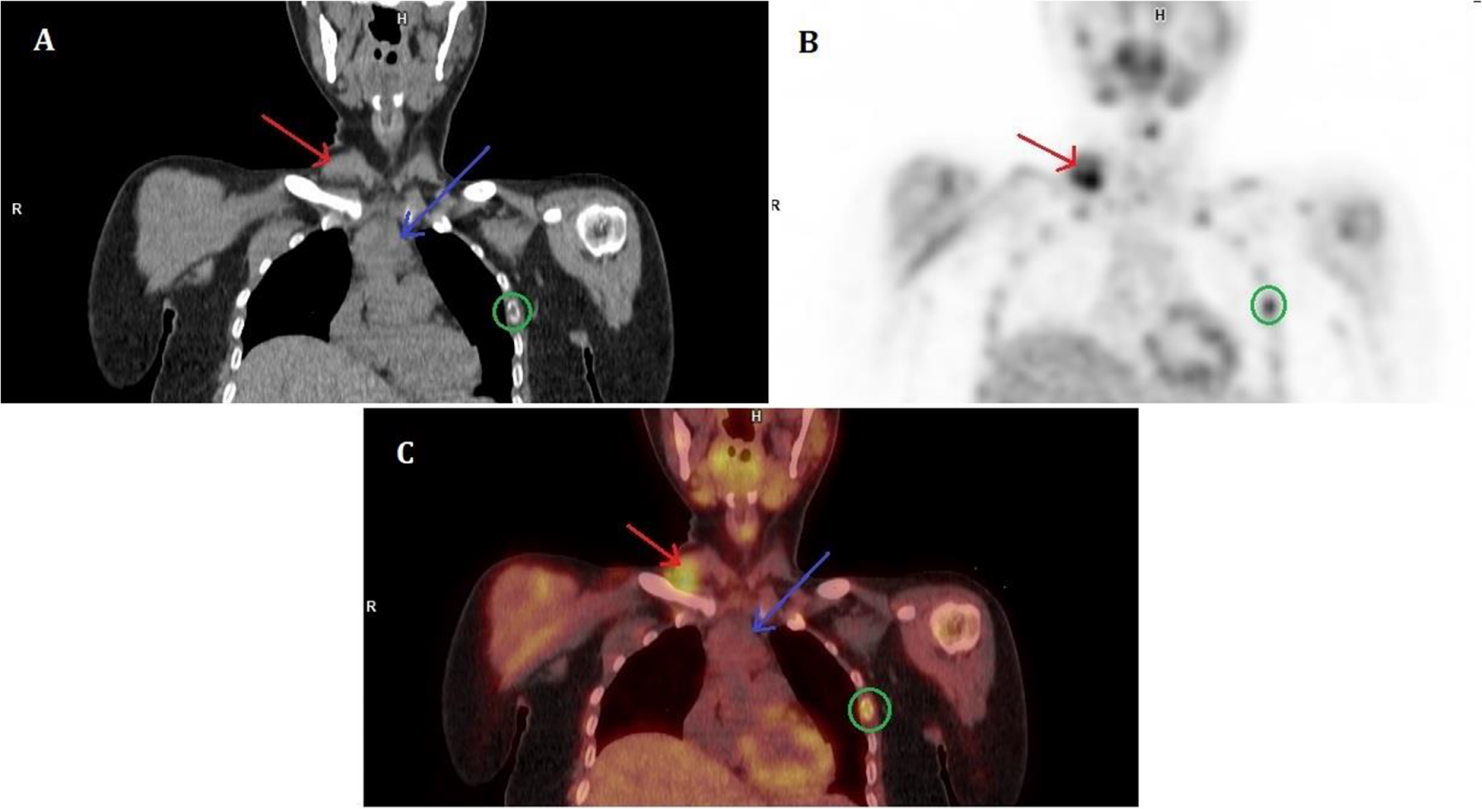
^18^F-FDG PET/CT: **A** coronal computed tomography image, **B** coronal PET images, and **C** fused PET/CT coronal image. There are FDG-avid right supraclavicular lymph node (red arrows), left fourth rib lytic lesion (green circles), and non-FDG-avid large anterior mediastinal soft tissue mass (blue arrows)

**Fig. 4:**
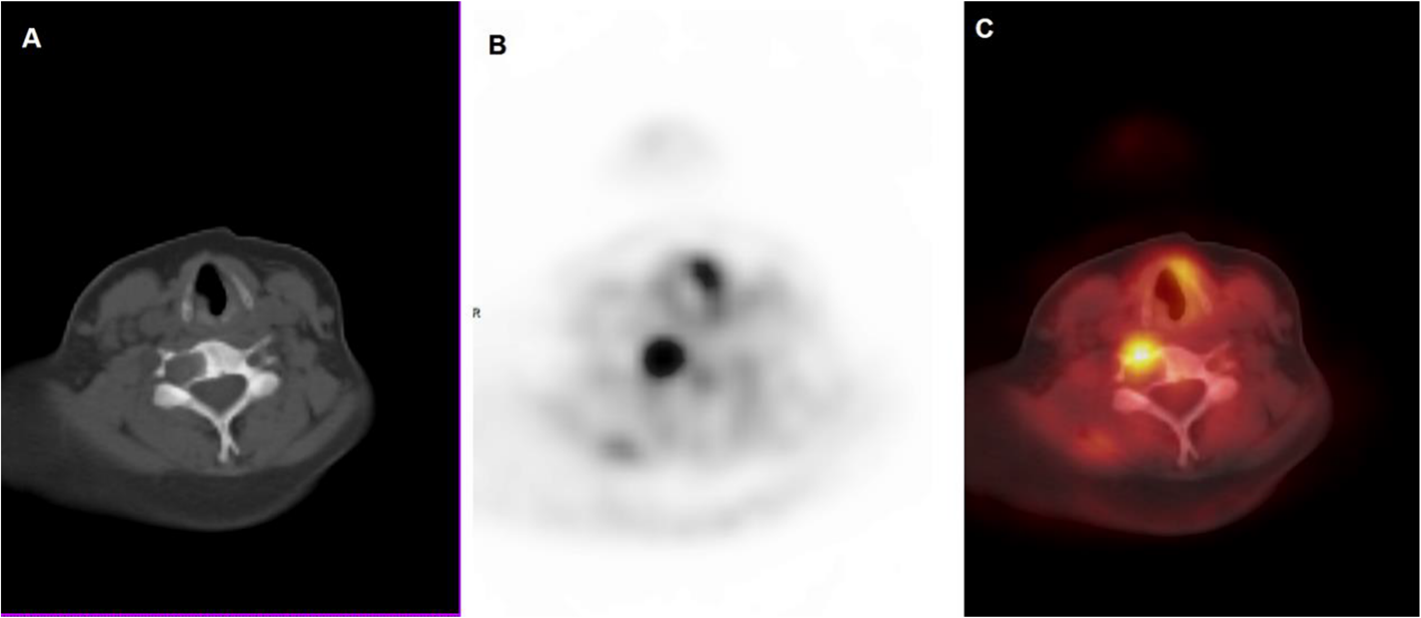
^18^F-FDG PET/CT: **A** axial computed tomography image showed a lytic lesion in the right side of C6 vertebral body, **B** axial PET images, and **C** fused PET/CT axial image showed FDG-avid lytic lesion in the right side of C6 vertebral body

**Fig. 5:**
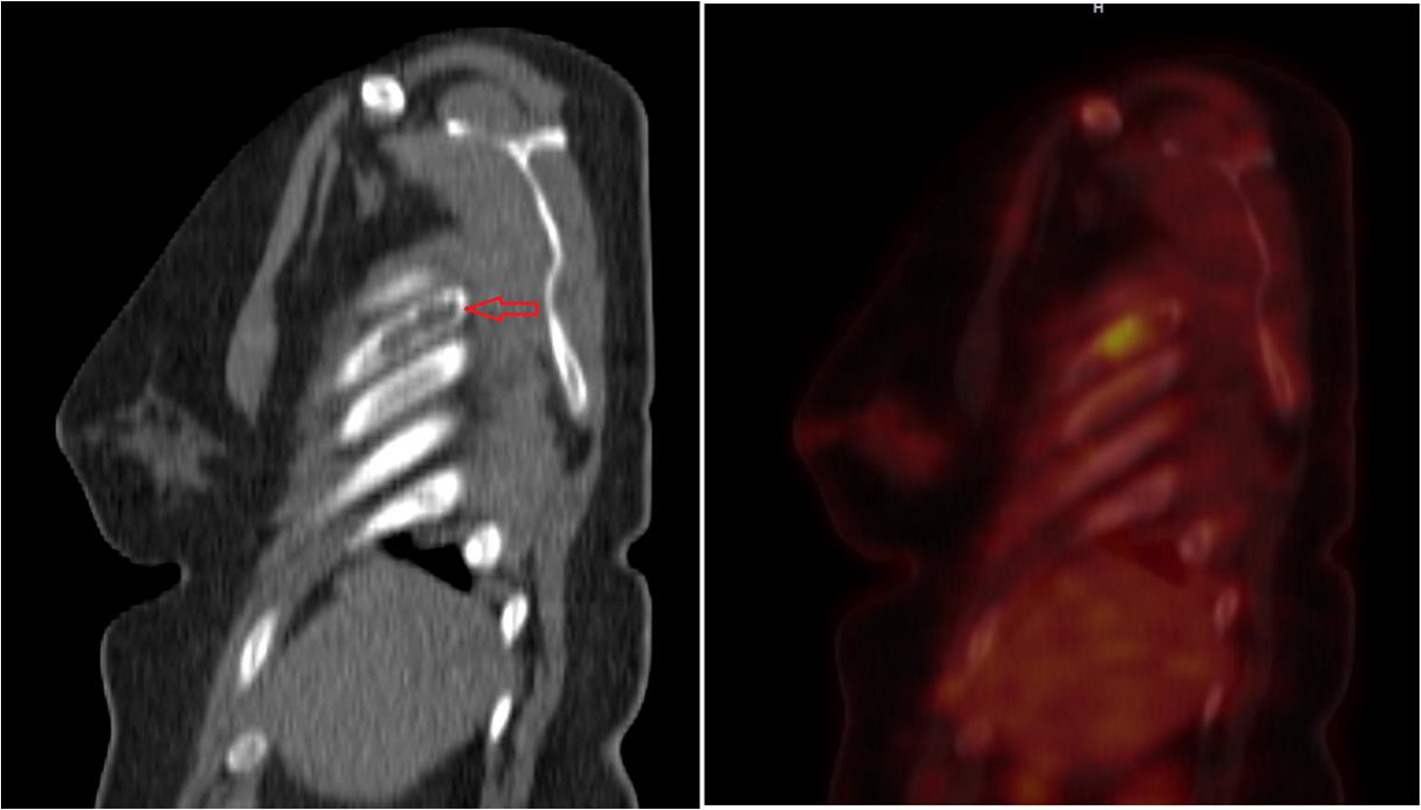
Sagittal computed tomography image and sagittal fused ^18^F-FDG PET/CT image: There is FDG-avid lytic lesion in lateral curvature of the left fourth rib

Whole-body fluorine-18-2-fluoro-2-deoxy-d-glucose positron emission tomography/computed tomography (PET/CT) (^18^F-FDG PET/CT) was done to assess the metabolic status of the right supraclavicular lymph nodes, mediastinal mass, and skeletal lesions. ^18^F-FDG PET/CT showed hypermetabolic FDG-avid right supraclavicular lymph node, suggesting metastatic thyroid carcinoma and hypermetabolic multiple lytic bone lesions with diffusely increased bone marrow metabolic activity in the axial and peripheral skeleton (Figs. 3, 4, and 5).

The right supraclavicular lymph node appeared metabolically active but non-RAI-avid, suggesting dedifferentiated features of the metastatic thyroid lesions.

Tc-99m MIBI-avid superior mediastinal lesion was non-FDG-avid, raising a possibility of an ectopic parathyroid mass with multiple brown tumors. However, with the patient’s history of thyroid malignancy, thyroid skeletal metastases could not be entirely excluded.

**Fig. 6:**
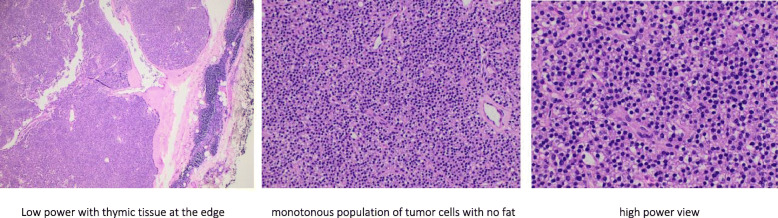
Histopathological findings: Sections show a hypercellular tumor composed mostly of round to polygonal cells with round monotonous/isometric nuclei and moderate eosinophilic granular cytoplasm. The cells are arranged in solid sheets and occasionally form small nests and pseudo-follicles. No intervening adipose tissue is seen. A remnant of thymic tissue appears at one edge. The overall feature perfectly fits a parathyroid adenoma

Right supraclavicular lymph node tru-cut biopsy revealed metastatic thyroid carcinoma. The patient underwent right neck dissection and mediastinal mass excision. Intra-operative PTH level declined from 863.7 to 100ng/ml. Final histopathological examination of the specimen revealed that the right supraclavicular lymph nodes were metastatic papillary thyroid carcinoma while the superior mediastinal mass was a parathyroid adenoma confirming the provisional impression of the ^18^F-FDG PET/CT (Fig. 6).

**Fig. 7:**
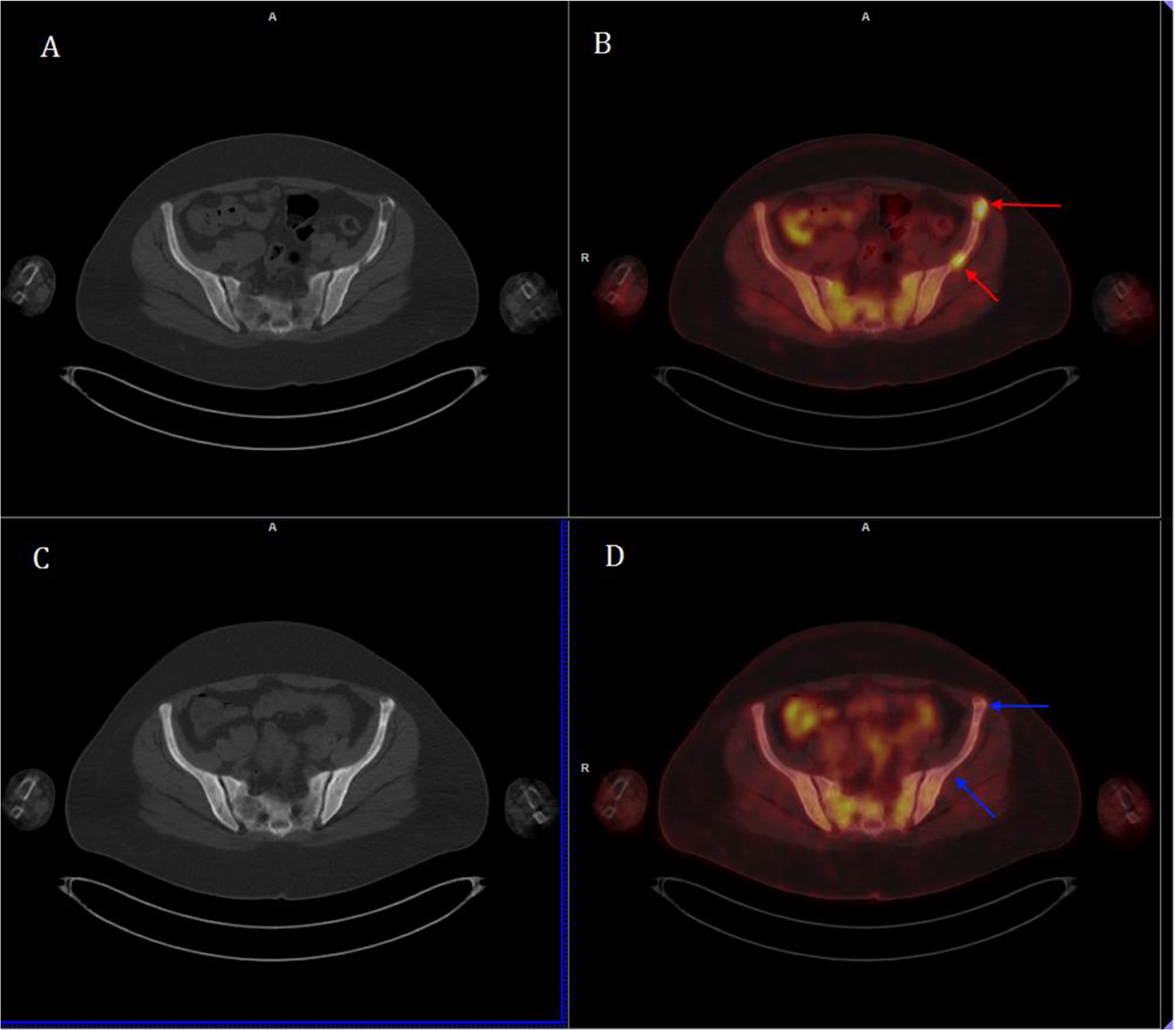
Preoperative and postoperative axial CT and fused ^18^F-FDG PET/CT images: **A**, **B** (preoperative) images show hypermetabolic left iliac lytic bone lesions (red arrows) that show complete metabolic resolution in the postoperative ^18^F-FDG PET/CT images (**C**, **D**) (blue arrows)

**Fig. 8:**
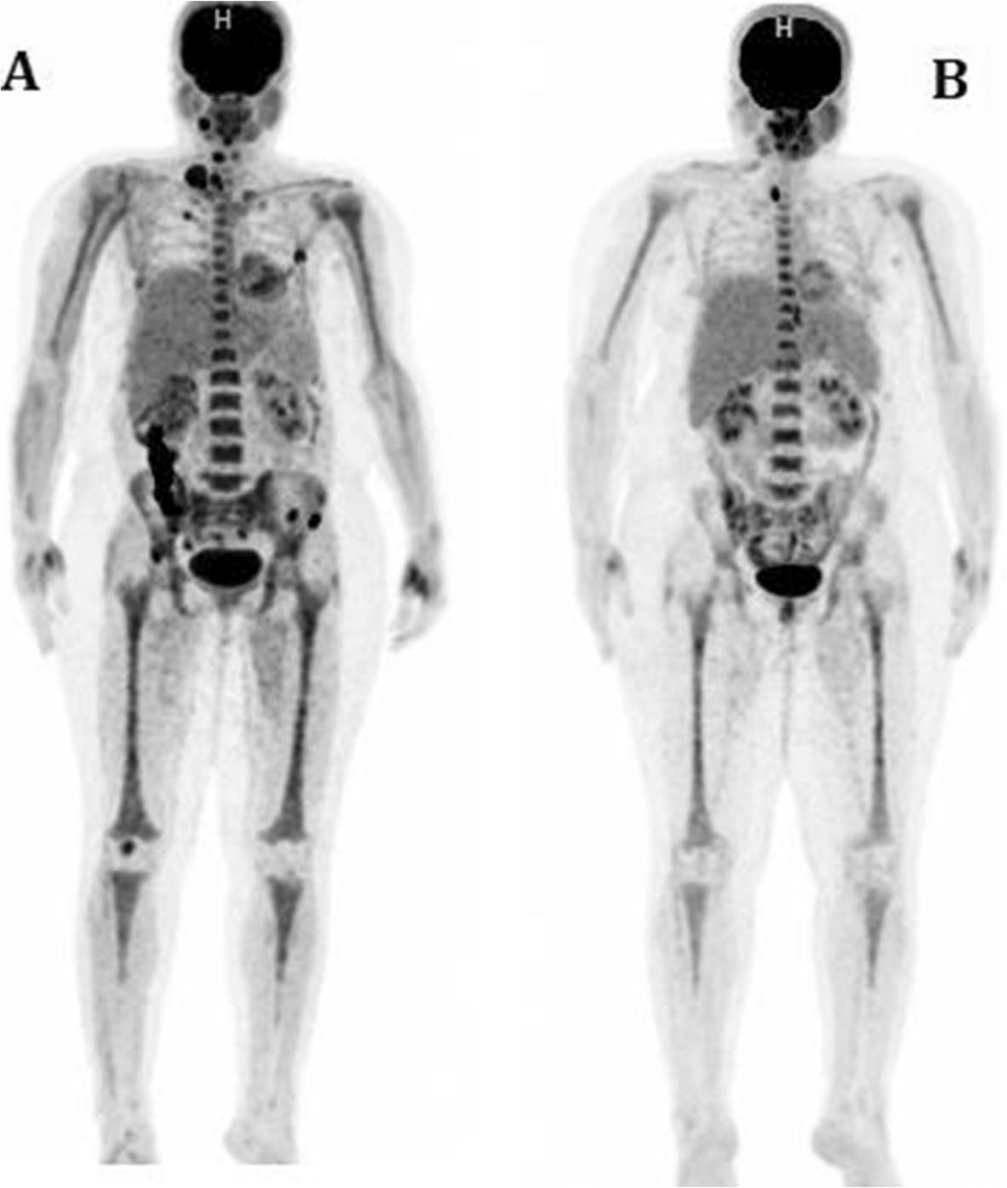
Pre- and postoperative maximum intensity projection images of ^18^F-FDG PET/CT: FDG-avid multiple skeletal lesions in the preoperative FDG PET/CT scan maximum intensity projection image (**A**) that shows complete metabolic resolution in the postoperative ^18^F-FDG PET/CT MIP image (**B**)

Ten months later, ^18^F-FDG PET/CT follow-up scan showed the resolution of all the previous FDG-avid skeletal lesions with some sclerotic changes, supporting the initial PET/CT supposition of brown tumors (Figs. 7 and 8 and Table 1).

**Table 1:** Summary of the main nuclear imaging findings and the assumptive diagnosis

Imaging modality	Findings	Assumptive diagnosis	Definitive diagnosis (pathology)
Whole-body iodine scan	Negative for any RAI-avid lesions		
^99m^Technetium sestamibi	^99m^Technetium sestamibi-avid right supraclavicular and mediastinal lesions	Ectopic parathyroid adenomas	Ectopic mediastinal parathyroid adenoma and metastatic papillary thyroid carcinoma of the right supraclavicular lymph node
Whole-body ^18^F-FDG PET/CT scan	FDG-avid right supraclavicular lymph node as well as multiple lytic bone lesions with diffusely increased bone marrow metabolic activity in the axial and peripheral skeleton	The right supraclavicular lymph node appears metabolically active but non-RAI-avid suggesting a possibility of having dedifferentiated features of the metastatic thyroid lesions. The ^99m^Tc MIBI-avid superior mediastinal lesion was non-FDG-avid raising a possibility of ectopic parathyroid mass with multiple brown tumors	The mediastinal lesion: ectopic parathyroid adenoma The right supraclavicular lymph node: metastatic papillary thyroid carcinoma
Postoperative follow-up whole-body ^18^F-FDG PET/CT scan	Complete metabolic resolution of all the previous FDG-avid skeletal lesions with some sclerotic changes	Supporting the previous assumption of ectopic parathyroid mass with multiple brown tumors	

## Discussion

Bone lytic lesions are common in patients with primary hyperparathyroidism. High levels of parathormone, excreted by the patients’ parathyroid glands, cause the resorption of their skeletal system. This bone resorption can lead to one of the primary hyperparathyroidism complications, brown tumors, where bones show localized fibrotic and cystic bony changes [2]. As skeletal manifestations of hyperparathyroidism, brown tumors occur in less than 2% of all patients with any form of hyperparathyroidism, which can be easily misdiagnosed as a malignant neoplasm, and it represents a real challenge for clinicians in the differential diagnosis [2].

This case presents a patient with a history of thyroid carcinoma who had multiple osteolytic lesions. Such history can easily be accepted as metastasis of a thyroid origin. Using different nuclear medicine imaging modalities and analyzing the difference in metabolic and functional status of the different etiologies, brown tumors were presented as a possibility.

Generally, the radiological feature similarities of brown tumors and lytic bone metastases or other bone tumors may cause a dilemma in diagnosis. A single brown tumor can be easily confused with a solitary bone cyst, aneurysmal bone cyst, giant cell tumor, or even a giant cell reparative granuloma. On the other side, the differential diagnosis of multiple brown tumors includes osteolytic metastasis, multiple myeloma, or multiple bone cysts [6].

In this case, the multiple osteolytic lesions were noniodine-avid but FDG-avid. However, the mediastinal soft tissue mass was Tc-99m MIBI-avid but non-FDG-avid, raising the possibility for those FDG-avid multiple osteolytic lesions to be brown tumors in association with these findings the Tc-99m MIBI-avid mediastinal soft tissue mass representing a parathyroid adenoma. In addition to that, the supraclavicular lymph node was FDG-avid but non-RAI-avid; this finding raised the possibility of a metastatic lymph node with dedifferentiated features in light of the rising sTg level. A meta-analysis of the 18F-FDG PET/CT’s diagnostic accuracy in patients presented with elevated sTg and negative whole-body scan post-thyroidectomy reported a good diagnostic accuracy of these methods with pooled sensitivity and specificity values of 88.5% and 84.7%, respectively [7, 8].

Differentiated thyroid cancer cells take up the radioiodine by expressing the sodium–iodide symporter. However, as cells dedifferentiate and the disease becomes more aggressive, their ability to concentrate iodine is lost (leading to reduced radioiodine uptake), and cellular glucose metabolism activates (with increased FDG uptake). This pattern was defined as the “flip-flop phenomenon” by Feine [8].

Radiologically, brown tumors appear as lytic lesions with well-defined borders; their differential diagnosis includes primarily bone metastasis, amyloid cysts, chondroma, aneurysmal bone cyst, osteosarcoma, and GCT or myeloplax tumors [4]. Brown tumors show increased FDG accumulation on ^18^F-FDG PET/CT [9].

The most common sites for brown tumors are facial bones, jaw, pelvis, ribs, femurs, and other long bones [4]. Pathologically, brown tumors represent a reparative cellular process with a localized accumulation of fibrosis tissue and giant cells that can replace bone and even produce osseous expansion. Histopathological findings include osteoclastic resorption with cavities filled with fibrous tissue, giant cells, hemosiderin deposits, and macrophages [10].

After the resection of parathyroid adenoma, the follow-up ^18^F-FDG PET/CT showed re-mineralization of the brown tumors with osteoblastic transformation due to reversal of the parathyroid hormone effect, and all showing complete metabolic resolution. While there was no histological proof, comparing the pre-surgical and post-surgical 18F-FDG PET/CT scan findings, the post-surgical decrease in calcium and PTH levels supports the assumption that they were only brown tumors and not bone metastases.

This case report emphasizes the impact of the multimodality approach in reaching an adequate diagnosis when the patient presented in different concurrent entities and facilitated receiving the proper treatment plan without exposing her to more aggressive treatment. However, our case report has some limitations as no whole-body or single-photon emission tomography was acquired in the MIBI scan to adequately correlate the findings of the PET scan in the brown tumor lytic bone lesions.

## Conclusions

Brown tumors can be differentiated from bone metastases. When presented concurrently, parathyroid adenoma and parathyroid carcinoma can be diagnosed. Such diagnosis requires the proper employment and careful interpretation of the different metabolic and functional imaging modalities. Ultimately, an appropriate management approach can be reached.

## Data Availability

Not applicable.

## Abbreviations

ALP: Alkaline phosphatase
CT: Computed tomography
^18^F-FDG: Fluorine-18-2-fluoro-2-deoxy-d-glucose
GCT: Giant cell tumor
MRI: Magnetic resonance imaging
PET/CT: Positron emission tomography/computed tomography
RAI: Radioactive iodine (I-131) therapy
S.PTH: Serum parathyroid hormone
sTg: Stimulated serum thyroglobulin
Tc-99m MIBI: Technetium-99m-sestamibi
